# Essential oils expose diverse targets on non-enveloped ScV-L-A totivirus

**DOI:** 10.1080/13880209.2025.2555815

**Published:** 2025-09-11

**Authors:** Algirdas Valys, Živilė Strazdaitė-Žielienė, Algirdas Mikalkėnas, Jurga Būdienė, Enrika Celitan, Saulius Serva, Elena Servienė

**Affiliations:** a Laboratory of Genetics, State Scientific Research Institute Nature Research Centre, Vilnius, Lithuania; b Department of Biochemistry and Molecular Biology, Institute of Biosciences, Vilnius University, Vilnius, Lithuania; c Laboratory of Chemical and Behavioral Ecology, State Scientific Research Institute Nature Research Centre, Vilnius, Lithuania

**Keywords:** Natural products, EOs, antivirals, RNA polymerase, viral particles, GC-MS, TEM

## Abstract

**Context:**

The spread of novel viruses substantiates the need for alternative antiviral agents. Plant-based extracts, such as essential oils (EOs), are highly relevant due to their generally human-friendly nature and broad spectrum of bioactive properties. Most EO antiviral activity is targeting enveloped viruses. *Orthototiviridae* family yeast virus ScV-L-A offers a novel, safe, non-enveloped virus model system for antiviral substance evaluation.

**Objective:**

This study aimed to investigate the antiviral efficacy of EOs and their constituents against non-enveloped ScV-L-A totivirus.

**Materials and methods:**

The composition of EOs was determined using GC-MS. Native ScV-L-A viral particles were prepared from yeast cells *via* cesium chloride gradient ultracentrifugation. The antiviral effect of EOs and their principal components was evaluated by following the synthesis of radio-labeled viral transcripts. TEM was employed to investigate the impact of target substances on ScV-L-A capsid integrity.

**Results:**

Tested EOs inhibited viral RNA polymerase activity in both liquid and vapor phases. Citral-rich EOs exhibited the strongest antiviral action, with lemon myrtle EO possessing the lowest half-maximal inhibitory concentration and highest timewise efficacy. Coriander and mandarin EOs showed the lowest polymerase-inhibiting capacity. EOs were more efficient compared to the action of single compounds. All EOs except for that of mandarin, dominated by limonene, were more effective in the liquid rather than the vapor phase. Tea tree and mandarin EOs were found to damage ScV-L-A capsid structure.

**Discussion and conclusion:**

This study highlighted the efficacy of EOs in targeting non-enveloped viruses and revealed their potential for sustainable control of viral infection.

## Introduction

In recent years, alternative antiviral agents have been intensely sought as a response to emerging novel viruses. Plant-based substances and extracts, such as essential oils (EOs), garner great interest due to their broad spectrum of bioactive properties, eco- and human-friendly nature (de Sousa et al. 2023; He et al. 2023). EOs are hydrophobic liquid concentrates of plant secondary metabolites, extracted from various plant flowers, leaves, fruits, seeds, bark, and other parts. EOs are usually comprised of lipophilic, low molecular weight, volatile molecules—mostly terpenes, their oxidated derivatives—terpenoids, and phenylpropanoids (Sadgrove et al. 2022). The composition of EOs varies significantly, primarily depending on the plant species and the specific parts used for extraction. Plant growth conditions, age, cultivar, extraction method used, and other factors can also influence the content of EOs (da Silva et al. 2021). Usually, no more than 2 to 5 dominant components comprise the majority of an EO and primarily define its biological properties, as other components are present in significantly smaller amounts (de Sousa et al. 2023). Sometimes the biological activity of EOs cannot be ascribed to any of the major components, and only their combination determines the biological properties of an EO (Wani et al. 2021). As most EO constituents are volatile, these substances have the potential to be applied for virus inactivation in their gaseous phase; however, until now, very few studies have investigated the antiviral activity of their vapor (Usachev et al. 2013; Madia et al. 2022; Başer et al. 2023; Şakalar and Ertürk 2023).

It has been shown that some EOs and their primary constituents possess significant antiviral activity against both DNA and RNA viruses (Wani et al. 2021; Sulaiman et al. 2022). The general mechanism of EOs towards viruses can be regarded as denaturants, considering the chemical structure of their components (Reichling 2022). While in most cases the exact molecular mechanisms of inhibition of viral replication remain unknown, several modes of action have been suggested. It has been determined that EOs and their components can disrupt virion morphology (Lai et al. 2012; Madia et al. 2022), mask external virion structures (Gilling et al. 2014), prevent viral entry into cells (Li et al. 2013), inhibit viral protein functioning (Feriotto et al. 2018; Abdullah et al. 2023), and viral gene expression (Chen et al. 2015; Wu et al. 2016), subsequently preventing viral infection. The majority of research investigating the antiviral effect of EOs has been conducted against enveloped human-infecting viruses, and the compounds found in EOs are presumed to primarily exert their antiviral influence by damaging the integrity of the viral lipid envelope (Reichling 2022). The lipid envelope-lacking viruses persist on the surfaces for a longer time and are more virulent than enveloped ones; thus, they are less susceptible to inactivation by different bioactive agents, including EOs (Reichling 2022; Narula et al. 2024). Therefore, the development of formulations that would be effective against non-enveloped viruses yet safe for humans is in high demand.

In this study, we selected yeast *Saccharomyces cerevisiae* virus L-A (ScV-L-A), belonging to the *Orthototiviridae* family, as a non-enveloped virus model system for evaluation of the antiviral properties of EOs and their principal components. ScV-L-A possesses a dsRNA genome, encompassing two open reading frames—the first encodes the major coat protein Gag, and the second—an RNA-dependent RNA polymerase, expressed as a Gag-Pol fusion protein due to a −1 ribosomal frameshift event that occurs sparingly during translation (Dinman et al. 1991; Schmitt and Breinig 2002). The ScV-L-A capsid is approximately 40 nm in diameter, possesses *T* = 1 icosahedral symmetry, and is comprised of 120 copies of capsid protein, one or two of these being the Gag-Pol fusion variant, arranged in asymmetric dimers (Naitow et al. 2002). ScV-L-A viral particles (VPs) are not densely packed, what facilitates enhanced mobility of the template, and contain 18 Å diameter openings at the five-fold symmetry axes, which are postulated to serve for the entry of nucleotide triphosphates and the exit of viral transcripts (Naitow et al. 2002; Grybchuk et al. 2022).

There are several advantages of ScV-L-A selection as a model system for the antiviral testing of EOs. First and foremost, the structure of the ScV-L-A nucleocapsid is quite simple, comprised of only the capsid protein, polymerase, and the dsRNA genome; therefore, it was expected to be straightforward to determine the precise target of antiviral substances in comparison to more complex viruses. Secondly, the purification of intact virions from the native host prepares the genome-synthesizing structures available for in-depth analysis of polymerase activity. Thirdly, the replication cycle of ScV-L-A lacks an extracellular phase, thus making the risk of viral contamination inconsequential; even more, these viruses are not pathogenic to humans and therefore pose no threat to the health of the researchers involved.

In the current study, the antiviral activity of selected EOs and their main components was investigated for the first time, using the non-enveloped *Orthototiviridae* family ScV-L-A as a model system. An extensive set of EOs, including those of lemon myrtle (*Backhousia citriodora* F. Muell., Myrtaceae), lemongrass (*Cymbopogon citratus* (DC.) Stapf, Poaceae), palmarosa (*Cymbopogon martini* var. *motia* Bruno, Poaceae), lavender (*Lavandula angustifolia* Mill., Lamiaceae), coriander (*Coriandrum sativum* L., Apiaceae), mandarin (*Citrus reticulata* Blanco, Rutaceae), lemon verbena (*Aloysia citriodora* Ortega ex Pers., Verbenaceae) and tea tree (*Melaleuca alternifolia* Cheel, Myrtaceae), along with chemically synthesized compounds, representing main EO constituents (citral, geraniol, geranyl acetate, linalool, linalyl acetate, *α*-pinene, limonene, terpinen-4-ol and 1,8-cineole) were investigated. The chemical composition of the tested EOs was determined by gas chromatography-mass spectrometry (GC-MS), the antiviral activity of EOs and their main components was evaluated in direct contact (liquid) and vapor phase by targeting viral RNA polymerase and the impact on the structure of VPs was observed by transmission electron microscopy (TEM).

## Materials and methods

### Yeast strain and growth medium

*Saccharomyces cerevisiae* strain M437 [L + M-] (*wt*, HM/Hm [kil-0]) carrying ScV-L-A-lus (a subspecies of ScV-L-A) (isolated from wine by Naumov and Naumova 1973) was used for the purification of VPs. YPD medium (1% yeast extract, 2% peptone (Liofilchem, Roseto degli Abruzzi, Italy, cat. no. 611005 and 611701, accordingly), 2% dextrose (Biolife, Milan, Italy, cat. no. 4125012)) was used for yeast propagation.

### Essential oils and their principal components

**Figure 1.**
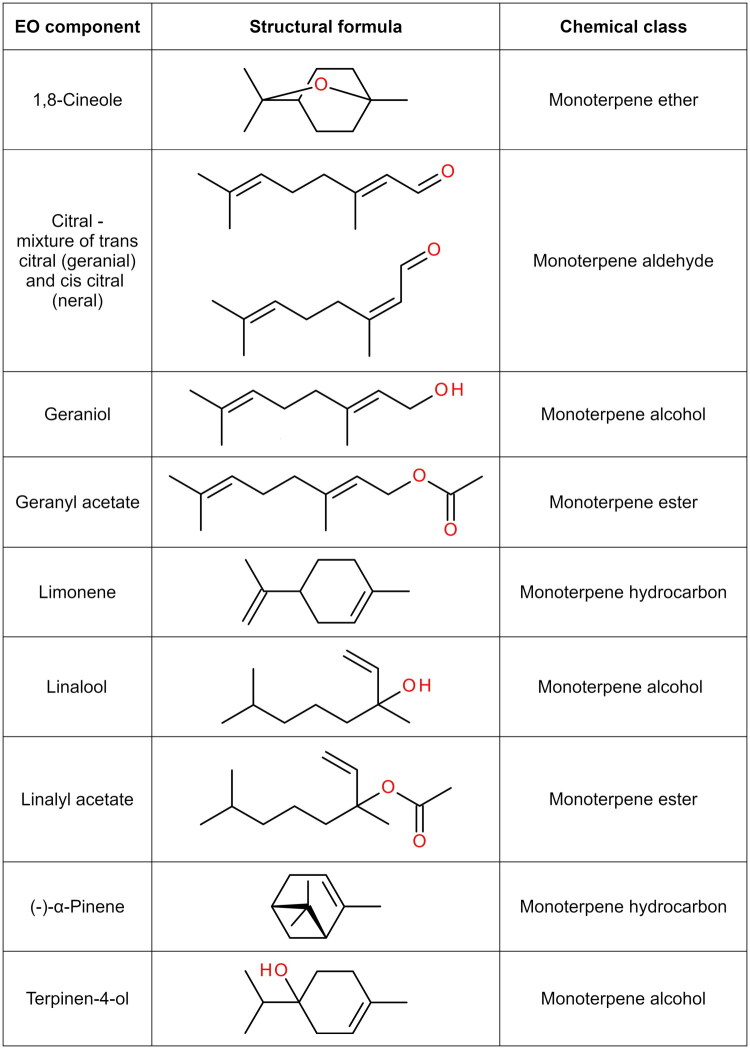
Chemical structures of major components of EOs tested in this study.

Liquid EO preparations were purchased from JSC “Kvapų namai” (Vilnius, Lithuania) (https://aromata.lt/en) from 2022 to 2023. Different parts of aromatic plants grown in various countries were used for the preparation of purchased EOs: leaves and twigs of lemon myrtle derived from Australia (batch no. B666021; cat. no. 47732193), leaves and stems of lemon grass*—*from India (batch no. LEMGC1221KYE; cat. no. 47732339), whole herb of palmarosa—from India (batch no. LOT 1222/5; cat. no. 47732254), flowering parts of lavender—from France (batch no. LOT 0033083; cat. no. 47733619), coriander seeds—from France (batch no. LOT 230086; cat. no. 47750227), mandarin peels—from Italy (batch no. LOT 0032457; cat. no. 47733657), lemon verbena leaves—from Spain (batch no. LOT 4056; cat. no. 47731752), and tea tree leaves—from South Africa (batch no. TEAO0423EEO; cat. no. 47731868). Chemically synthesized principal components of EOs: geraniol (cat. no. 4988.1) with a purity ≥90%, citral (*E*/*Z*-isomer mixture, cat. no. 5937.1), limonene (racemic mixture, cat. no. 7516.1), terpinen-4-ol (racemic mixture, cat. no. 6309.1) with a purity ≥95%, (-)-*α*-pinene (cat. no. 5647.1), 1,8-cineole (cat. no. 7244.1) with a purity ≥98% and pure linalool (racemic mixture, cat. no. 3992.1) were purchased from Carl Roth (Karlsruhe, Germany); linalyl acetate (racemic mixture, cat. no. 49599) with a purity ≥97% and geranyl acetate (cat. no. 45896) with a purity ≥99% were purchased from Sigma-Aldrich (Buchs, Switzerland). The structural formulas of these compounds are presented in Figure 1.

### Essential oil chemical composition analysis determined by gas chromatography-mass spectrometry

The chemical composition of all the tested EOs was determined by GC-MS. EOs (10 µL of each) were dissolved in 1 mL of a pentane and diethyl ether (Sigma-Aldrich, Buchs, Switzerland, cat. no. 1.00882 and 1.00931, accordingly) (1:1) mixture, and 1 µL of the prepared solution was injected into the GC-MS system. Shimadzu GC/MS-Q2010 PLUS, interfaced to Shimadzu GC-MS-QP2010 ULTRA mass spectrometer, fitted with a non-polar Rxi-5MS (30 m × 0.25 mm × 0.25 µm) capillary column, was used for analysis. Mass spectra in electron impact mode were generated at 70 eV, 0.97 scans per second, a mass range of 33–400 m/z. The oven temperature of the gas chromatograph was set at 50 °C (for 1 min) and then increased by 5 °C per minute to reach 160 °C, then held for 2 min and programmed to reach 250 °C at the increased rate of 10 °C/min and hold at the final temperature for 4 min. Helium with a flow rate of 1.0 mL/min was used as carrier gas. Detector and injector temperatures were 250 °C, and the ions source temperature was 220 °C. The qualitative analysis and identification of compounds were based on the comparison of time and retention indexes of the column, with corresponding data in the literature (Adams 2007), as well as computer libraries of mass spectra (using “GC/MS solution” v. 2.71 software from Shimadzu and Wiley and NIST). The identification of the compound was approved if mass spectra library data matched the computer data with a probability equal to 90% or above. The retention indexes were determined regarding retention times of a series of *n*-alkanes (C_7_–C_30_) with linear interpolation. The relative percentage of EO constituents was computed from the chromatogram peak areas without the correction factors. Principal Component Analysis (PCA) was performed on the online statistics platform statskingdom.com. The method was applied using a percentage of the eight EOs and thirteen variables (individual constituents with an amount ≥5% in at least one sample).

### Purification of viral particles

Yeast cells were grown in liquid YPD medium at 30 °C for 16 to 24 h. The pellet was collected by centrifuging at 5000 × g for 5 min at 4 °C and washed with distilled water. For the purification of VPs, about 2 g of cells were used. The yeast cell wall was eliminated using zymolyase as described in Dunn and Wobble (2001) and obtained spheroplasts were resuspended in AB buffer (20 mM Tris-HCl, pH 7.5 (AppliChem, Darmstadt, Germany, cat. no. A2264,1000), 50 mM KCl and 10 mM MgCl_2_ (Carl Roth, Karlsruhe, Germany, cat. no. P017.1 and 2189.1, accordingly)) containing 1% phenylmethylsulfonylfluoride (Sigma-Aldrich, Darmstadt, Germany, cat. no. P7626) and disrupted by vigorous vortexing with glass beads (Lukša et al. 2022). Glass beads were removed by centrifugation at 1000 × g for 10 min at 4 °C and crude cell debris was eliminated by 10 min of centrifugation at 10000 × g. The collected supernatant was supplemented by 0.25 volume of 25% PEG4000 (Sigma-Aldrich, Darmstadt, Germany, cat. no. 25322-68-3) in AB buffer with 2.5 M NaCl (Carl Roth, Karlsruhe, Germany, cat. no. HN00.2), incubated for 30 min on ice, and centrifuged at 15000 × g for 10 min at 4 °C. The pellet was washed with AB buffer containing 5% PEG4000 and 3 M NaCl and finally suspended in 220 μL of AB buffer. The samples were further purified by CsCl (Carl Roth, Karlsruhe, Germany, cat. no. 7878.1) density gradient (1.36 g/cm^3^) ultracentrifugation at 127,000 × g for 24 h (Lukša et al. 2022). The presence of VPs was confirmed by SDS-PAGE. Agarose gel electrophoresis was used for viral RNA detection. Selected fractions with the highest VP content were concentrated with Amicon Ultracel YM-100 centrifugal filter devices (Millipore, Bedford, MA, USA, cat. no. UFC5100) to a desired volume. The buffer solution was changed using Zeba^™^ Spin Desalting Columns, 7K MWCO (Thermo Fisher Scientific, Vilnius, Lithuania, cat. no. 89883) with 2× VPs storage solution (100 mM Tris-HCl, pH 7.6, 300 mM NaCl, 2 mM EDTA (Sigma-Aldrich, Darmstadt, Germany, cat. no. 10378-23-1)). After buffer exchange, glycerol (Penta, Prague, Czech Republic, cat. no. 14530-11000PE) was added to the solution of VPs up to 50% of the final concentration for long-term storage.

### Antiviral activity test in the liquid phase

Isolated VP suspension (5 µL, containing approximately 1 × 10^9^ VPs, as determined by approximating the number of viral genome copies through electrophoretic analysis) was mixed with an equal volume of EOs or their principal components, and incubated at room temperature (22 °C) for 1, 2, or 24 h. VPs mixed with an equal volume of 10% ethanol (Stumbras, Kaunas, Lithuania) were used as negative controls. To detect the half maximal inhibitory concentration (IC_50_), EOs and their principal components were sequentially diluted by half with 10% ethanol, 5 µl of the prepared dilutions were mixed with an equal volume of VPs and incubated at room temperature for 2 h. For the evaluation of residual RNA polymerase activity, an *in vitro* assay was carried out with affected and control samples. All experiments were performed in three biological replicates, and treatment efficiency is expressed as the mean value ± standard deviation.

### Antiviral activity test in the vapor phase

Isolated VP suspension (5 µL, containing approximately 1 × 10^9^ VPs) was diluted with 5 µL of DEPC-treated water (Thermo Scientific, Vilnius, Lithuania, cat. no. R0601) and placed at the bottom of a test tube, and 25 µL of EOs or their principal components were placed inside the lid of the test tube. The tube was closed, wrapped with parafilm, placed horizontally (to avoid direct contact between liquids), and incubated at room temperature for 1, 2, or 24 h. Test tubes containing 25 µL of 10% ethanol in the insides of their lids were used as negative controls. The residual RNA polymerase activity was assessed in affected and control samples. Each experiment was carried out in three biological replicates, and the treatment efficiency is expressed as means ± standard deviations.

### In vitro assay for determining viral RNA polymerase activity

Minor modifications were made to the method previously described by Ribas and Wickner (1996). The 20 µl reaction mixture contained 50 mM Tris-HCl (pH 7.6), 10 mM MgCl_2_, 20 mM NaCl, 5 mM KCl, 10 mM 2-mercaptoethanol (Calbiochem, San Diego, CA, USA, cat. no. 4449), 0.5 mM each of UTP, CTP, ATP and GTP (ThermoFischer Scientific, Vilnius, Lithuania, cat. no. R0481), 10 nM [*α*-^32^P] ATP (Revvity/PerkinElmer, Shelton, CT, USA, cat. no. BLU503H2-50UC), 1 U/µL RiboLock RNase Inhibitor (Thermo Fisher Scientific, Vilnius, Lithuania, cat. no. EO0382), 5% PEG6000 (Carl Roth, Karlsruhe, Germany, cat. no. 0158.1), 10 µL of VP suspension from an affected or control sample. The reaction mixture was incubated for 180 min at 30 °C; afterward mixed with STOP solution (85% formamide (Carl Roth, Karlsruhe, Germany, cat. no. P040.2), 0.06% bromphenol blue (Sigma-Aldrich, Buchs, Switzerland, cat. no. B0126), 25 mM EDTA, 1% SDS (Bio-Rad, Hercules, CA, USA, cat. no. 161-0301)) at a ratio 1:1 (v:v) and analyzed on a 7% polyacrylamide gel with 8 M urea (Carl Roth, Karlsruhe, Germany, cat. no. X999.3) and subjected to autoradiography. Electrophoretic images were analyzed using the software ImageJ (version 1.53k). The effect of EOs on RNA polymerase activity was determined by comparing the amount of synthesized radio-labeled RNA between control and affected samples.

### Transmission electron microscopy analysis

To observe any structural changes to the VPs following treatment with EOs or compounds, representing main constituents of EOs, TEM imaging was performed. 3 µL of a sample was poured on carbon-coated copper grids (Agar Scientific, Stansted, UK, cat. no. AGS162-4), dried with filter paper, and washed with 3 µL of sterile water for 10 s. The particles were stained with 3 µL of 2% aqueous uranyl acetate (SPI Supplies, West Chester, PA, USA cat. no. 6159-44-0) solution for 10 s, dried with filter paper, and additionally air-dried for 1 min. Electron microscopy of stained particles was performed using a Morgagni 268 (D) transmission electron microscope (FEI, Hillsboro, OR, USA).

### Statistical analysis

Regression analysis was performed to estimate the IC_50_, and mean IC_50_ values along with their standard deviations were calculated from the results of three independent experiments. Calculation of the 95% confidence intervals (CIs) for IC_50_ values was performed according to Hazra (2017), using the t-distribution. One-way analysis of variance (ANOVA) and Tukey’s post-hoc test were used to evaluate the significance of differences between IC_50_ values. The differences were considered statistically significant at *p* < 0.05. All analyses were performed using GraphPad Prism 10.5.0 (CA, USA).

## Results

### Chemical composition of the tested essential oils

**Figure 2.**
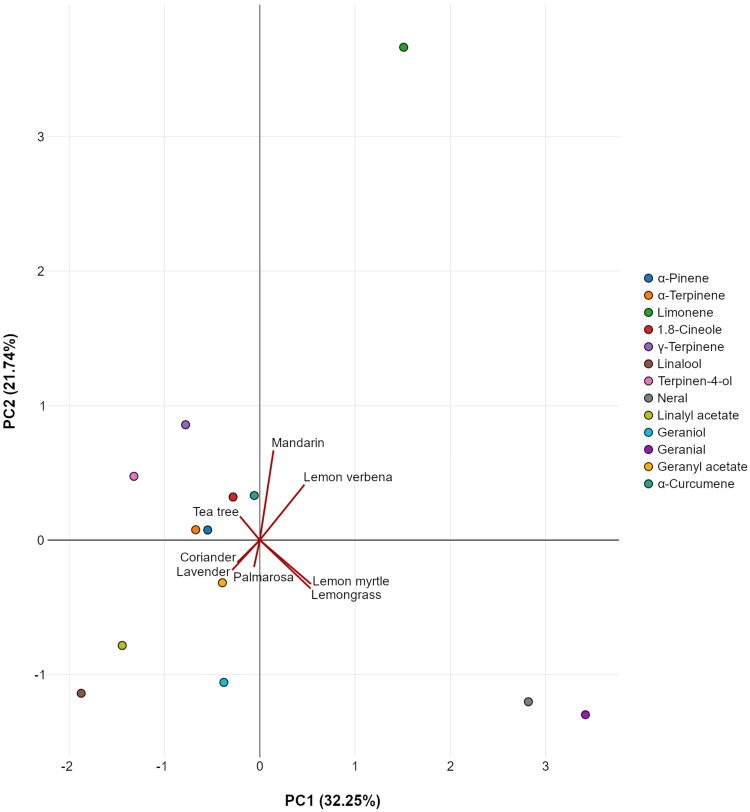
PCA analysis of the tested EOs based on their components, which constitute 5% of at least one of the EOs.

GC-MS was employed to analyze the composition of EOs. Table 1 presents the main constituents of the tested EOs, and supplementary Table S1 lists their full composition. The EOs of this study were divided into four groups based on the chemical class of their dominant components and PCA-based clustering (Figure 2, Table 1). The first group included lemon myrtle and lemon grass EOs, characterized by the highest content of citral. The second group was comprised of palmarosa and lavender EOs, which are dominated by alcohols geraniol and linalool, respectively, and their corresponding acetate esters; as well as coriander EO, which is dominated by alcohol linalool. The third group consisted of mandarin and lemon verbena EOs, which possessed a high amount of hydrocarbon limonene. The only EO of the fourth group was that of tea tree with alcohol terpinen-4-ol as the primary component.

**Table 1.** Main components (%, including constituents with quantity ≥1.0 % in at least one of the EOs) of EOs of I group – lemon myrtle and lemon grass; II group – palmarosa, lavender, and coriander; III group – mandarin and lemon verbena; IV group – tea tree.

No	Compound	RI_Lit._/RI_Exp._	Group I		Group II			Group III		Group IV
			Lemon myrtle	Lemongrass	Palmarosa	Lavender	Coriander	Mandarin	Lemon verbena	Tea tree
1	***α*-Pinene**	939/939		0.19		0.14	**7.67**	2.87	1.37	2.79
2	Camphene	954/955		1.03	0.01	0.12	1.09	0.01	0.02	0.01
3	Sabinene	975/974		0.01		0.03	0.37	0.27	3.19	0.16
4	*β*-Pinene	979/978		0.01		0.07	0.64	2.14	0.23	0.76
5	6-methyl-5-hepten-2-one	985/985	0.26	0.98	0.02				4.34	
6	Myrcene	990/990			0.42	0.66	1.13	2.34	0.75	0.85
7	***α*-Terpinene**	1018/1017				0.13	0.04	0.32	0.11	**9.07**
8	*para*-Cymene	1024/1024	0.03			0.19	0.53	0.66	0.12	3.82
9	**Limonene**	1029/1030		0.27	0.17		2.63	**67.06**	**22.96**	1.37
10	**1.8-Cineole**	1031/1031				0.90			**5.37**	**5.98**
11	(*Z*)-*β*-Ocimene	1039/1040		0.25	0.42	3.88	0.01		0.05	
12	(*E*)-*β*-Ocimene	1050/1050		0.14	1.62	3.30	0.01	0.02	1.42	
13	***γ*-Terpinene**	1059/1059			0.01	0.12	**6.82**	**19.5**	0.29	**19.23**
14	4-Nonanone	1053/1071		1.14						
15	Terpinolene	1088/1088		0.04	0.02	0.05	0.60	1.03	0.08	3.23
16	**Linalool**	1096/1096	0.50	0.95	2.31	**24.61**	**67.94**	0.12	0.91	0.09
17	3-acetoxy-1-octene	1102/1103				1.81				
18	Camphor	1141/1145				0.34	4.07		0.09	
19	(*Z*)-Isogeranial	1164/1165	1.62	0.87					0.26	
20	Lavandulol	1169/1169				1.14				
21	Borneol	1169/1169		0.3		1.14	0.17			
22	**Terpinen-4-ol**	1177/1178		0.04		4.22	0.13	0.09	0.28	**40.88**
23	(*E*)-Isogeranial	1180/1180	3.04	1.72					0.52	
24	*α*-Terpineol	1188/1188		0.19		0.40	0.21	0.23	1.49	3.12
25	**Neral**	1238/1239	**42.35**	**33.82**	0.25				**9.80**	
26	**Linalyl acetate**	1257/1256				**38.16**			0.02	
27	**Geraniol**	1252/1257	1.47	**8.44**	**70.80**		1.50			
28	**Geranial**	1268/1267	**48.10**	**39.62**					**12.56**	
29	Lavandulyl acetate	1288/1285				4.55				
30	**Geranyl acetate**	1381/1380	0.23	3.63	**17.76**	0.44	3.89		1.34	
31	(*E*)-Caryophyllene	1419/1419	0.08	1.42	1.93	4.46	0.07	0.22	2.95	0.34
32	Aromadendrene	1440/1441								1.06
33	*α*-Humulene	1454/1454	0.01	0.40	0.14	1.85		0.02	0.32	0.07
34	***α*-Curcumene**	1480/1481							**6.77**	
35	Bicyclogermacrene	1500/1500	0.06			0.04			1.34	0.17
36	γ-Cadinene	1512/1513		1.07	0.03	0.17			0.26	0.02
37	*δ*-Cadinene	1523/1523	0.02	0.20	0.02			0.03	0.43	1.08
38	Spathulenol	1578/1578	0.04						2.18	0.06
39	Caryophyllene oxide	1583/1583	0.03	0.33	0.19	0.31		0.01	2.61	
40	Geranyl hexanoate	1725/1725			1.50					
	**Total**		**97.84**	**95.34**	**97.62**	**93.23**	**99.52**	**96.94**	**84.43**	**94.16**

RILit: Kovat’s indices for non-polar column DB-5 taken from Adams (2007); RIExp: Kovat’s indices determined experimentally on the non-polar column Rxi-5MS (Restek. USA); bolded compounds are those whose amounts were greater than 5% in at least one of the oils.

Regarding the first group of EOs, the major constituents of lemon myrtle EO were two isomers of citral—geranial (48.1%) and neral (42.35%); among the minor components prevailed (*E*)-isogeranial (3.04%), (*Z*)-isogeranial (1.62%), and geraniol (1.47%) (Table 1). Lemon grass EO was characterized by a high level of geranial (39.62%) and neral (33.82%); moderate levels of geraniol (8.44%) and geranyl acetate (3.63%) were observed. Besides the two isomers of citral, 12 other constituents were common among these two oils, including linalool, geraniol, and others (Table S1).

From the second group, palmarosa EO was dominated by geraniol (70.80%) and geranyl acetate (17.76%) and also possessed a minor amount of linalool (2.31%) (Table 1). The principal components of lavender EO included linalyl acetate (38.16%) and linalool (24.61%), as well as a moderate level of terpinen-4-ol (4.22%). The composition of coriander EO was dominated by alcohol linalool (67.94%); it also contained a substantial amount of monoterpene hydrocarbons, *α*-pinene (7.67%), *γ*-terpinene (6.82%), and limonene (2.63%), as well as geranyl acetate (3.89%). Linalool and geranyl acetate, as well as 6 other components were found to be shared by the EOs of the second group (Table S1).

The EO of mandarin, belonging to the third group, was dominated by monoterpene hydrocarbon limonene (67.06%) and γ-terpinene (19.50%); a moderate level of *α*-pinene (2.87%) was also detected (Table 1). The most abundant components in lemon verbena EO were limonene (22.96%) and citral (consisting of geranial (12.56%) and neral (9.80%)); while other monoterpene hydrocarbons and alcohols were detected in minor quantities. It was determined that 26 components were common between mandarin and lemon verbena EOs, the primary ones being limonene and *α*-pinene, whereas others were present only in trace amounts in either one or both oils (Table S1).

The major component of tea tree EO, the only one from the fourth group, was monoterpene alcohol terpinen-4-ol (40.88%). It also contained a notable amount of hydrocarbons *γ*-terpinene (19.23%), and *α*-terpinene (9.07%), and alcohols 1,8-cineole (5.98%) and *α*-terpineol (3.12%) (Table 1).

### Utilizing the ScV-L-A for the antiviral analysis of essential oils

**Figure 3.**
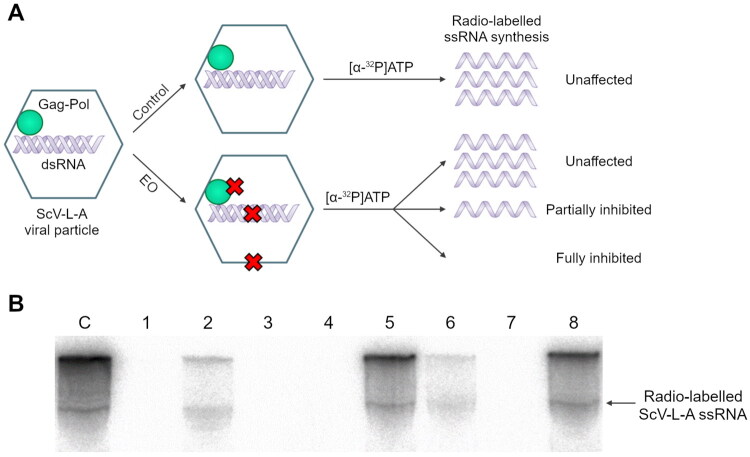
Schematic representation of possible action modes of EOs on the synthesis of new viral RNA (A) and PAGE visualizing the effects of EOs and single compounds on the inhibition of RNA polymerase activity after 2 h exposure in liquid (l) and vapor (v) phases (B). C – untreated control, 1 – palmarosa EO (l), 2 – palmarosa EO (v), 3 – lemon myrtle EO (l), 4 – lemon myrtle EO (v), 5 – limonene (l), 6 – limonene (v), 7 – citral (l), 8 – citral (v).

**Figure 4.**
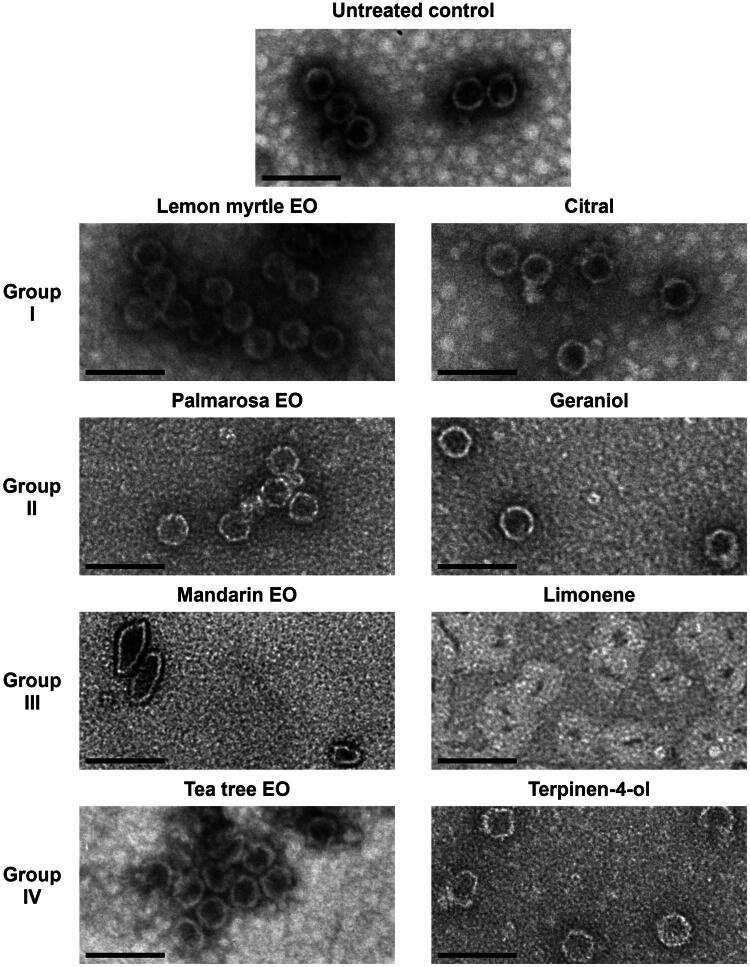
TEM analysis of the effect of EOs and single compounds, representative of each of the four groups, on ScV-L-A capsid structure. VPs were exposed to the vapor of the tested substances for 2 h. Scale bar – 100 nm.

An isogenic *S. cerevisiae* yeast strain carrying ScV-L-A was cultivated and used for VP purification by CsCl gradient ultracentrifugation. Resulted fractions were analyzed by SDS-PAGE analysis (Fig. S1A) and agarose gel electrophoresis (Fig. S1B). Fractions containing most RNA were pooled and prepared for antiviral testing and polymerase reactions, as expected to possess a native virus structure containing viral genomic dsRNA. The isolated VPs were used to study the inhibitory action of EOs in liquid and vapor phases. The antiviral effect of EOs and pure components was evaluated by testing the incorporation of radio-labeled nucleotides, which directly reflects the yeast viral RNA polymerase activity. Residual RNA polymerase activity was measured by comparing the ScV-L-A ssRNA band intensity between the control and tested samples (Figure 3). EOs and their constituents could impart the functionality of RNA polymerase by interacting with all three of the ScV-L-A VP structural components—the RNA polymerase itself, the capsid proteins, and the dsRNA genome (Figure 3A). As shown in Figure 3B, EOs and their main components can induce complete (Figure 3B-1, 3, 4, 7) or partial inhibition of viral RNA polymerase (Figure 3B-2, 6) as well as remain neutral toward the RNA polymerase activity (Figure 3B-5, 8).

### Impact of essential oils and their main components on viral particles in liquid and vapor phases

Isolated ScV-L-A VPs were treated *in vitro* by EOs and some of their primary components in liquid and vapor phases for 1, 2, or 24 h at room temperature. Residual viral RNA polymerase activity was evaluated by observing the incorporation of radio-labeled nucleotides into synthesized RNA molecules.

It was found that in the liquid phase at 50% concentration, citral-rich lemon myrtle and lemongrass EOs were able to inhibit viral RNA polymerase activity by 100 and 90.51%, respectively, in 1 h (Table 2). The analysis of half-maximal inhibiting concentrations (IC_50_) confirmed lemon myrtle EO to be more effective than lemongrass—after 2 h of exposure, it showed an IC_50_ value of 4.53% (95% CI: 4.05–5.01%), which differed significantly and was about three times lower than that of lemongrass EO (IC_50_ value of 15.54%, 95% CI: 15.22–15.86%) (Table 3). Pure citral at the highest concentration displayed only partial inactivation of 47.85% in 1 h, and nearly complete polymerase inhibition was achieved after 2 h (94.80%) (Table 4). In the liquid phase, the IC_50_ of citral was reached at 15.40% concentration (95% CI: 14.84–16.24%), similar to lemongrass. In the vapor phase, 1 h of exposure to lemon myrtle EO resulted in almost complete inactivation of viral RNA polymerase activity (93.78%). However, the effect of lemongrass EO was distinctly weaker—RNA polymerase activity was reduced by 22.21% after 1 h of exposure, and by 92.60% after 2 h. On the other hand, the effect of citral vapor, even after 2 h, was negligible (5.23%), and complete RNA polymerase activity inhibition was registered only after 24 h (Table 4).

**Table 2.** The effect of EOs in their liquid and vapor phases on ScV-L-A RNA polymerase activity, expressed in % of RNA polymerase activity inhibition as mean ± SD.

EOs	Liquid phase			Vapor phase		
	1h	2h	24h	1h	2h	24h
Lemon myrtle	100.00 ± 0.00	100.00 ± 0.00	100.00 ± 0.00	93.78 ± 5.29	100.00 ± 0.00	100.00 ± 0.00
Lemongrass	90.51 ± 7.26	96.20 ± 5.37	100.00 ± 0.00	22.21 ± 8.66	92.60 ± 6.90	98.84 ± 1.65
Palmarosa	61.26 ± 5.60	100.00 ± 0.00	100.00 ± 0.00	10.38 ± 3.89	61.49 ± 1.68	100.00 ± 0.00
Lavender	77.13 ± 6.96	83.47 ± 3.44	100.00 ± 0.00	10.14 ± 3.30	15.49 ± 9,39	93.47 ± 9.24
Coriander	19.89 ± 8.27	69.13 ± 2.37	91.19 ± 7.83	13.14 ± 4.58	22.62 ± 9.56	100.00 ± 0.00
Mandarin	10.07 ± 8.22	38.77 ± 9.74	56.43 ± 7.53	66.40 ± 5.13	99.37 ± 0.90	100.00 ± 0.00
Lemon verbena	91.75 ± 11.66	100.00 ± 0.00	100.00 ± 0.00	98.13 ± 1.76	100.00 ± 0.00	100.00 ± 0.00
Tea tree	42.55 ± 2.54	98.76 ± 1.75	100.00 ± 0.00	3.70 ± 3.63	23.70 ± 8.66	100.00 ± 0.00

**Table 3.** The IC_50_ values of tested EOs and their primary components, expressed in % (v/v).

EO or terpene	IC_50_, %	95% CI, %	Group
Lemon myrtle EO	4.53 ± 0.19	4.05–5.01	a
Tea tree EO	14.39 ± 0.23	13.94–14.95	b
Citral	15.40 ± 0.62	14.84–16.24	b
Lemongrass EO	15.54 ± 0.28	15.22–15.86	b
Palmarosa EO	28.26 ± 1.51	24.52–32.00	c
Terpinen-4-ol	28.68 ± 1.36	25.30–32.06	c
Lavender EO	31.31 ± 3.22	23.30–39.32	cd
Geraniol	34.50 ± 2.51	28.27–40.73	d
Lemon verbena EO	41.24 ± 1.30	38.01–44.47	e
Coriander EO	46.28 ± 0.48	44.47–48.09	f

Listed in ascending order are only those substances that possessed an IC_50_ value lower than 50%. substances denoted by common group letters indicate no significant difference (*p* < 0.05, tukey’s test) between the IC_50_ of those substances.

**Table 4.** The effect of terpenes in their liquid and vapor phases on ScV-L-A RNA polymerase activity, expressed in % of RNA polymerase activity inhibition as mean ± SD.

Terpenes	Liquid phase			Vapor phase		
	1h	2h	24h	1h	2h	24h
Citral	47.85 ± 3.77	94.80 ± 4.15	100.00 ± 0.00	4.31 ± 3.06	5.23 ± 5.98	100.00 ± 0.00
Geraniol	80.67 ± 1.84	98.74 ± 1.78	100.00 ± 0.00	28.47 ± 2.60	96.34 ± 4.10	100.00 ± 0.00
Geranyl acetate	18.82 ± 6.11	22.13 ± 2.46	89.77 ± 9.75	2.69 ± 2.89	33.54 ± 9.78	94.83 ± 7.31
Linalool	25.21 ± 9.34	41.04 ± 4.21	100.00 ± 0.00	13.03 ± 6.17	27.37 ± 8.54	100.00 ± 0.00
Linalyl acetate	32.49 ± 4.05	35.32 ± 3.29	86.44 ± 6.22	12.73 ± 9.55	26.17 ± 6.78	96.53 ± 2.46
Terpinen-4-ol	96.00 ± 3.08	99.37 ± 0.89	100.00 ± 0.00	3.38 ± 4.78	6.33 ± 5.23	100.00 ± 0.00
Limonene	8.35 ± 6.11	22.20 ± 6.70	56.59 ± 10.55	32.39 ± 9.42	44.71 ± 10.28	99.53 ± 0.66
α-pinene	9.04 ± 11.94	9.10 ± 6.58	90.34 ± 11.33	0.86 ± 1.22	22.54 ± 7.68	54.88 ± 6.99
1,8-cineole	37.84 ± 4.06	45.63 ± 6.56	89.75 ± 7.34	13.04 ± 6.83	27.09 ± 8.29	67.01 ± 10.08

After 1 h of exposure in the liquid phase, the EOs of the second group (palmarosa, lavender, and coriander) at 50% concentration caused partial inhibition of viral RNA polymerase activity, with lavender EO displaying the strongest (77.13%) and coriander EO the weakest (19.89%) effect (Table 2). On the other hand, 2 h of exposure was sufficient to induce complete inhibition by palmarosa EO, while the inhibition caused by lavender and coriander EOs remained partial (83.47 and 69.13%, respectively). The detected lower IC_50_ value of palmarosa EO (28.26%, 95% CI: 24.52–32.00%) compared to those of lavender (31.31%, 95% CI: 23.30–39.32%) and coriander (46.28%, 95% CI: 44.47–48.09%) confirmed it having the strongest viral RNA polymerase inhibition potential of EOs from the second group, although the difference between IC_50_ values of palmarosa and lavender EOs was not statistically significant (Table 3). In the vapor phase, all three EOs induced a weak inhibition of RNA polymerase activity of about 10% in 1 h, and after 2 h, palmarosa EO was the only one to exert a notably stronger effect (61.49%). However, 24 h was sufficient to induce a complete or nearly complete RNA polymerase inactivation by all EOs of the second group in both liquid and vapor phases (Table 2).

Geraniol, which constitutes the majority of palmarosa EO, at maximal concentration displayed a slightly stronger effect than the EO, executing the inhibition of viral RNA polymerase activity of 80.67% after 1 h in the liquid phase and almost complete inhibition after 2 h in both the liquid and vapor phases (98.74 and 96.34% respectively) (Table 4). This is somewhat in contrast to the fact that the IC_50_ of geraniol (34.50%, 95% CI 28.27–40.73%) was significantly greater than that of palmarosa EO (Table 3). Contrarily, geranyl acetate seemed weaker than palmarosa EO, inducing inhibition of only about 20% after 1 and 2 h in the liquid phase and acting with similar efficacy in the vapor phase. On the other hand, linalool, constituting about a quarter of lavender EO and two-thirds of coriander EO, exerted a somewhat weaker effect than the EOs in the liquid phase, inhibiting viral RNA polymerase activity only by 41.04% after 2 h, but displayed a comparable effect as the EOs in the vapor phase (Table 2). The efficacy of linalyl acetate was very close to that of linalool, the major difference being that after 24 h, it did not fully inhibit viral RNA polymerase activity, causing inhibition of 86.44% and 96.53% in liquid and vapor phases, respectively. The second most abundant component of coriander EO *α*-pinene was notably less effective in the liquid phase than both coriander EO and linalool, and even more so in the vapor phase, inactivating the viral RNA polymerase by only 54.88% after 24 h (Tables 2 and  4).

Lemon verbena, of the third group of EOs, exerted a strong effect by inhibiting RNA polymerase activity after 1 h by 91.75 and 98.13% in the liquid and vapor phases, respectively, leading to full inhibition after 2 h in both phases (Table 2). Surprisingly, it was determined that this EO had a relatively high IC_50_ value of 41.24% (95% CI: 38.01–44.47%) (Table 3). Mandarin EO, on the other hand, exerted a very weak effect in the liquid phase, at 50% concentration, inhibiting RNA polymerase activity by only 56.43% after 24 h. However, it displayed a stronger effect in the vapor phase, causing inhibition of 66.40 and 99.37% after 1 and 2 h, respectively (Table 2). Limonene, which comprises the majority of mandarin EO, displayed a comparable effect as the EO in the liquid phase and acted slightly weaker in the vapor phase, inactivating the RNA polymerase by only 44.71% after 2 h (Table 4).

Tea tree EO inhibited viral RNA polymerase activity by 98.76% after 2 h in the liquid phase at maximal concentration and by only 23.70% in the vapor phase in the same period, while 24 h were sufficient to induce complete inhibition (Table 2). Its primary component, terpinen-4-ol, exerted a stronger effect in the liquid phase after 1 h (96.00%) but displayed a similar efficacy as the EO otherwise (Table 4). However, the IC_50_ value of tea tree EO (14.39%, 95% CI: 13.94–14.95%) was the second lowest of all the substances tested and two-fold lower than that of terpinen-4-ol (28.68%, 95% CI: 25.30–32.06%), with the difference being statistically significant (Table 3). 1,8-cineole, a secondary component of tea tree EO, induced a notably weaker inhibition of the viral RNA polymerase after 2 h in the liquid phase (45.63%) and failed to induce complete inhibition in the vapor phase even after 24 h (67.01%) (Table 4).

### Effect of essential oils and their components on viral particle morphology

To investigate the effect of exposure to EOs and their main components on ScV-L-A VP morphology, TEM was employed. Since high concentrations of EO components impair TEM imaging, VPs were exposed to the vapor of selected substances. A representative EO from each of the four groups and their most abundant components were selected for TEM analysis. From the first group, citral and lemon myrtle EO were selected, due to this EO having a stronger effect on ScV-L-A RNA polymerase activity than lemongrass EO (Table 2). Palmarosa EO and geraniol were chosen to represent the second group for the same reason. Mandarin EO and limonene were selected as the representatives of the third group since lemon verbena EO displayed a closer viral RNA polymerase inhibition pattern to citral-rich EOs from the first group. Tea tree EO and its main component, terpinen-4-ol, were the only choices from the fourth group.

Based on visual qualitative analysis of TEM images, it was determined that lemon myrtle and palmarosa EOs as well as their major constituents did not induce any noticeable morphological alterations on ScV-L-A VPs (Figure 4). However, in the sample affected by mandarin EO, disfigured VPs could be seen—some were flattened and elongated, others smaller, with indents or otherwise irregularly shaped. The limonene-affected sample was abundant in globular structures larger in size than ScV-L-A VPs but with much smaller cores, suggesting a strong denaturing of capsid proteins. Contrarily, tea tree EO and terpinen-4-ol did not seem to induce such strong alterations, but in samples affected by these substances, a greater abundance of VPs with partially disintegrated capsid structure could be seen than in the untreated control or samples affected by substances belonging to the first or second groups.

## Discussion

EOs and their respective constituents are promising natural alternatives for the treatment of viral infections and disinfection of surroundings, both used independently and in combination with other antiviral measures (Ma and Yao 2020; de Sousa et al. 2023). While the complex variation-prone composition of EOs can pose challenges to their application in practice, they nevertheless appear to be a more natural choice compared to isolated terpenes. In some cases, EOs have been shown to possess a greater selectivity index (defined as the ratio of 50% cytotoxic concentration and IC_50_) than their pure constituents, therefore being safer to use (Garozzo et al. 2009; Astani et al. 2010; Ćavar Zeljković et al. 2022). In this study, we tested the antiviral activity of 8 EOs and 9 of their pure principal components in their liquid and vapor phases on ScV-L-A, a dsRNA genome-possessing non-enveloped yeast virus, as a model system.

GC-MS analysis of the EOs of this study revealed their composition to be similar to those determined by other researchers. Citral constituted over 90% of our lemon myrtle EO and about 70% of our lemongrass EO, the trans isomer geranial being the dominant one in both cases. These data align with previously published results, stating that citral usually comprises about 80–90% of lemon myrtle EO and 70–80% of lemongrass EO, with the abundance of geranial prevailing over neral (Torres Neto et al. 2022; Nagata et al. 2024). Geraniol combined with geranyl acetate accounts for close to 90% of our palmarosa EO, with the amount of geraniol being several times greater than that of its acetate ester, as reported by other researchers (Dangol et al. 2023). Nearly 60% of the lavender EO used in this study was comprised of linalool and linalyl acetate, the latter being more abundant. This is similar to reports observing the combined amount of these substances in lavender EO to be between 50 and 70%; although which of these two compounds is the prevailing one tends to vary (Ciocarlan et al. 2021). The most abundant component of coriander EO was linalool, making up nearly 70% of the EO, and the second most abundant was *α*-pinene—this is also in accordance with the findings of other researchers (Gastón et al. 2016). Limonene is the dominant compound in our tested mandarin EO, constituting close to 70% of it, and corresponds to previously described mandarin EO compositions with limonene comprising about 50–80% (Lu et al. 2013; Bava et al. 2021). Our lemon verbena EO also possessed quite a large amount of limonene as well as citral, both accounting for about 20% of the oil each. Ebadi et al. (2017) report similar levels of these constituents in lemon verbena EOs, while other sources claim a somewhat lower amount of limonene and a higher citral content, ranging between 30 and 50% (Ocazionez et al. 2010; Hematian Sourki et al. 2021). As for the tea tree EO, it was dominated by terpinen-4-ol, making up about 40% of the EO, and *γ*-terpinene, accounting for half as much. Most researchers report a composition similar to ours, with terpinen-4-ol levels reaching approximately 40% and a similar variety of secondary components (Lu et al. 2013; Elmi et al. 2019).

The antiviral properties of some plant extracts, the EOs of which were tested in this study, have already been investigated. Lemongrass, lavender, lemon verbena, and tea tree EOs have been shown to possess antiviral efficacy against some enveloped viruses, including herpes simplex virus 1 (HSV1), dengue virus, yellow fever virus, influenza A virus (IFVA), vesicular stomatitis virus and coronaviruses (Minami et al. 2003; Astani et al. 2010; Ocazionez et al. 2010; Gómez et al. 2013; Rosmalena et al. 2019; Abou Baker et al. 2021; Madia et al. 2022; Romeo et al. 2022; Torres Neto et al. 2022). As for non-enveloped viruses, it has been shown that lemongrass EO inactivates murine norovirus (MNV) (Gilling et al. 2014; Kim et al. 2017), and pre-treatment of cells with coriander extract partially inhibits its replication (Seo and Choi 2017). Tobacco mosaic virus (TMV) is known to be inactivated by mandarin and tea tree EOs (Lu et al. 2013), with the latter also having been shown to act antivirally against coxsackievirus (Iacovelli et al. 2023). To the best of our knowledge, no studies on the antiviral effect of palmarosa EO have been published to date.

Owing to structural and functional differences, distinct viral species are expected to exhibit differing sensitivities to certain EOs. Regardless, our results on the relative antiviral potencies of the tested EOs against ScV-L-A follow published data on other viruses. Torres Neto et al. (2022) observed the IC_50_ value of lemongrass EO against SARS-CoV-2 delta variant pseudovirus to be over a hundred times lower than that of tea tree EO, and lemongrass EO is also reported to be more effective than tea tree EO against HSV1 (Minami et al. 2003) and TMV (Lu et al. 2013). Although in our case, the IC_50_ value of lemongrass was slightly higher than that of tea tree EO, the difference was not statistically significant, and lemongrass EO was more or equally as effective in inactivating ScV-L-A RNA polymerase at all timepoints (Tables 2 and 3). The results obtained by Minami et al. (2003) indicate that lavender EO exerts a similar antiviral efficacy against HSV1 as tea tree EO; again, this is comparable to our results, as in most cases the quantitative discrepancies between the yeast viral RNA polymerase inactivation caused by lavender and tea tree EOs were minor. On the other hand, Lu et al. (2013) have shown mandarin EO to be significantly more effective against TMV than tea tree EO in the liquid phase. Whereas in our study, mandarin EO exerted a notably weaker effect than tea tree EO in the liquid phase, whilst being more effective in the vapor phase.

The studies addressing the antiviral effect of EOs or their mixtures in the vapor phase are quite sparse. Several studies have shown the vapor of some EOs and their blends to exert a substantial antiviral effect in relatively large-volume spaces (Pyankov et al. 2012; Usachev et al. 2013; Mirskaya and Agranovski 2021; Başer et al. 2023; Şakalar and Ertürk 2023). Vimalanathan and Hudson (2014) as well as Madia et al. (2022) have employed an experimental setup similar to ours, placing the virus and EOs in separate ends of a microcentrifuge tube to investigate the antiviral efficacy of tea tree and other EOs against IFVA. In our study, mandarin EO and its primary component limonene were the only substances that displayed a much stronger effect in the vapor phase than in the liquid phase at all timepoints (Tables 2 and 4). While a few other substances showed a moderately stronger effect of their vapor at some timepoints, the prevailing trend was for the liquid phase to be more potent. Compound volatility is determined by the quantity and strength of intermolecular interactions, primarily influenced by the size and structure of the chemical backbone and the presence of functional groups (Atkins and Jones 2011). Therefore, hydrocarbon terpenes are usually more volatile than their oxygenated counterparts, as they cannot form hydrogen bonds, thus making their intermolecular interactions significantly weaker. This could in part explain the strong effect of mandarin EO and limonene in the vapor phase as compared to the liquid phase on ScV-L-A RNA polymerase activity. Hydrocarbon *α*-pinene, however, did not exhibit a stronger effect in the vapor phase compared to the liquid one, likely due to its more compact bicyclic structure decreasing volatility. It is also worth noting that the observed antiviral effect is determined not only by the rate at which these compounds evaporate and reach their targets but also by the intrinsic antiviral potential of the compounds themselves. The evaporation rate and subsequent efficacy of the vapor of EOs or their constituents are defined by environmental conditions, such as ambient temperature, humidity, and atmospheric pressure (Atkins and Jones 2011). While these parameters can be fixed in a laboratory setting, they vary greatly in real-life environments. Moreover, the method of facilitating EO vaporization, the volume of the space along with the rate of air flow strongly affect the concentration of EO vapor, further complicating its application in practice. Standardized methods for antiviral testing of EO vapor are still lacking and need to be developed to conduct thorough studies which would be mutually comparable. While the results provided and/or cited in this study are promising, there remains a crucial gap between laboratory and practice in this area.

In our study, EOs that displayed the strongest inactivation of ScV-L-A RNA polymerase, namely those of lemon myrtle, lemongrass, and lemon verbena, possessed a high amount of aldehyde citral (Table 1). A strong antiviral efficacy of citral is to be expected, as it is an α-β unsaturated aldehyde, and compounds belonging to this group are known to be chemically reactive and can damage both proteins and nucleic acids by forming various adducts (Moldogazieva et al. 2023). There is no clear tendency for EOs to display a stronger or weaker effect than their pure main components (Ma and Yao 2020). While Gilling et al. (2014) reported that citral was more effective than lemongrass EO against MNV, in our case, all three citral-possessing EOs exerted a stronger effect than citral itself against ScV-L-A RNA polymerase (Tables 2 and 4), pointing towards a possible synergistic effect caused by the admixture of secondary EO components. Interestingly, lemon verbena EO, only about 20% of which is made up of citral, displayed a similar efficacy in the liquid phase after 1 h as lemongrass EO, which has approximately three times as much citral, and a significantly stronger effect at the same time in the vapor phase. This indicates that citral is most likely not the only antivirally acting component in lemon verbena EO and provides further proof of the importance of secondary constituents on the sum effect of the mixture. Additionally, while displaying similar efficacy in the liquid phase at 50% concentration, the IC_50_ of lemongrass EO was almost threefold lower and significantly differed from the IC_50_ of lemon verbena EO.

In the context of other natural antiviral product research, a 50% concentration of EOs and terpenes is quite high and may be unreasonable in some areas of potential practical application. In our case, this concentration was initially dictated by the high concentration of VPs, which in turn was necessitated by the efficiency of ScV-L-A RNA polymerase and sensitivity of autoradiography, being required for proper visualization of new radio-labelled viral transcripts. Moreover, our results have shown that some EOs and single compounds display relatively weak efficacy, at least in the timespan of up to 2 h, even at such high concentrations. Thus, the extensive screening of the effect on RNA polymerase functionality at 50% concentration at different timepoints enabled us to compare substances with greatly varying potency of antiviral properties. While high concentrations of EOs do not pose much of a threat to human health when applied for surface or air disinfection, they would likely be harmful, considering topical application or oral consumption, in the case of at least some EOs (D’Amato et al. 2018). In this regard, more research is necessary with lower EO concentrations and various potential application methods, based on emulsions, nanoparticles or other technologies (da Silva et al. 2025). The low IC_50_ value-possessing EOs outlined in this study, namely those of lemon myrtle, tea tree, and lemongrass, are prime candidates for further investigation.

Out of the pure EO constituents, terpinen-4-ol, the primary component of tea tree EO, displayed the strongest effect in the liquid phase. Tea tree EO itself was only slightly less effective than terpinen-4-ol, whereas 1,8-cineole was weaker than both of them. Iacovelli et al. (2023) observed a similar tendency regarding the antiviral activity of these three substances against non-enveloped coxsackievirus. On the other hand, tea tree EO is much more efficacious than terpinen-4-ol against lipid envelope-possessing HSV1 (Astani et al. 2010), and TEM imaging has shown tea tree EO to be able to damage the lipid envelope of IFVA (Madia et al. 2022). Taken together, these results indicate that minor tea tree EO constituents, such as the relatively abundant hydrocarbon *γ*-terpinene, likely induce greater damage on the viral lipid envelope than terpinen-4-ol, although the ability of this compound to penetrate lipid membranes has also been recorded (Hąc-Wydro et al. 2017).

The observed inactivating effect of terpinen-4-ol against non-enveloped viruses proves that this compound can exert its antiviral activity in different ways other than damaging the integrity of the viral envelope. Indeed, molecular docking studies suggest that terpinen-4-ol and other terpene alcohols can form hydrogen bonds with functionally important amino acids of some viral proteins, and these interactions should be at least in part responsible for the antiviral effect (Li et al. 2013; Kulkarni et al. 2020; Abdullah et al. 2023). It is reasonable to assume that the terpene alcohols tested in this study likely inhibited the functioning of ScV-L-A RNA polymerase similarly. In this regard, the stronger inhibitory efficacy displayed by geraniol compared to linalool is understandable due to steric obstruction hindering the ability of linalool to form hydrogen bonds (Figure 1). The strong antiviral effect of terpinen-4-ol could be determined by its cyclic structure, which makes it more compact and hence likely able to insert itself into protein cavities inaccessible to geraniol and linalool. However, further *in silico* studies are required to draw any definite conclusions.

Considering the inhibition of viral RNA polymerase functioning by the corresponding acetate esters of linalool and geraniol, the efficacy of linalyl acetate was comparable to that of linalool, whereas geranyl acetate was found to be weaker than geraniol (Table 4). Given that both the VP storage media as well as the subsequent *in vitro* transcription reaction featured a pH of around 7.6, spontaneous hydrolysis of these esters was likely negligible. Therefore, the obtained results indicate that additional steric hindrance of the functional group of linalool in its acetate ester form does not substantially impair its antiviral functioning. In the case of geraniol, however, the acetate ester group obstructing the primary hydroxy group greatly reduced its activity, further proving its importance for the antiviral properties of geraniol.

Hydrocarbon terpenes, such as limonene, have been shown to act antivirally against both enveloped (Astani and Schnitzler 2014) and unenveloped (Lu et al. 2013) viruses, presumably in a similar manner to terpene alcohols, by damaging the integrity of the lipid envelope and/or interfering with viral protein functioning through Van der Waals interactions (Yang et al. 2011). Experimental evidence proves that oxygenated functional groups are not necessary for a terpene to possess strong antiviral properties, and in some cases, hydrocarbons may even be more efficacious than oxidized terpenes (Astani et al. 2010).

Interestingly, out of all the substances tested, hydrocarbon limonene induced the most pronounced disfigurement of ScV-L-A VP structure, despite inducing only moderate inactivation of viral RNA polymerase under the same conditions (Table 4 and Figure 4). Limonene-rich mandarin EO also caused strong morphological alteration of VPs, yet its effect was distinctly different than that of limonene. Whether mandarin EO-affected VP structures represent an intermediate state between native and limonene-damaged structures due to dilution of limonene in the EO by secondary components or if these components, acting together with limonene, are responsible for differential alteration of VP morphology remains unknown. Tea tree EO and terpinen-4-ol also showed the capability to disrupt the ScV-L-A capsid structure while only having a weak effect on the viral RNA polymerase activity after identical treatment. It is unclear whether this disruption is sufficient to inactivate the virus or expose the genomic dsRNA to the environment, and if these morphological alterations are irreversible. Conversely, lemon myrtle EO, citral, palmarosa EO, and geraniol did not induce any changes in VP structure despite strongly inactivating the ScV-L-A RNA polymerase. This is in contrast to the findings of Gilling et al. (2014), who found that citral-affected MNV virions greatly increased in size, suggesting that citral molecules had bound to and agglomerated on the surface of the capsid. Although it is not clear whether polymerase inhibition and disruption of particles are permanent, or how efficiently these compounds affect different viruses. Regardless, both VP structural integrity and viral polymerase functionality are necessary for the successful replication of viruses, and, taken together, these results underline the importance of utilizing different model systems and strategies for evaluating the antiviral efficacy of EOs and their components.

Due to the fact that ScV-L-A is not infective, it cannot naturally enter the host from the extracellular environment. In this study, RNA polymerase functionality has been evaluated as a proxy for virus viability due to its importance in the successful functioning of all RNA viruses. It is worth keeping in mind, however, that it does not directly reflect complete viral inactivation. Also, in some environment–virus–host systems, the inactivation of viral polymerases or other proteins induced by EOs or their constituents could be reversible—this is especially noteworthy in the case of terpene alcohols and other compounds, postulated to bind to proteins non-covalently. The complex mixture of components found in EOs, however, likely results in a broad array of targets affected and could overpower the reversibility of some of the damage induced on viruses. On the other hand, measuring the viral polymerase inactivation may not only overestimate but also underestimate the final antiviral effect, as was seen in the case of mandarin EO and limonene, which greatly damaged the VP structure. In this study, we measured the amount of produced viral transcripts without their further functional characterization. It could be that some EO components may impair viral polymerase functioning without inducing full inactivation, resulting in an increased deleterious mutation rate for the virus. More precise investigation of the mechanisms of EOs’ action towards the dsRNA genome of ScV-L-A and its utilization is currently underway.

## Conclusions

In conclusion, the present study provides new information regarding the antiviral efficacy of various EOs and their main components against the non-enveloped ScV-L-A totivirus. Detailed chemical composition of lemon myrtle, lemongrass, palmarosa, lavender, coriander, mandarin, lemon verbena, and tea tree EOs was revealed by applying GC-MS. It was demonstrated that citral-rich EOs possessed the greatest ScV-L-A RNA polymerase inactivation potential; tea tree and palmarosa EOs also exhibited a pronouncedly strong antiviral effect. Tested EOs were more effective while having direct contact in the liquid phase, except for mandarin EO, which showed greater activity in the vapor phase. It was determined that lemon myrtle, lemongrass, mandarin, and lemon verbena EOs were more effective than their pure compounds, while other EOs displayed a limited or similar efficacy as their major components. In contrast to the majority of previous studies focusing on the action of EOs on enveloped viruses, the present work has proven the ability of EOs to neutralize non-enveloped viruses. Moreover, the antiviral activity of palmarosa EO has been demonstrated for the first time in this work. Tea tree and mandarin EOs, along with their major constituents (terpinen-4-ol and limonene, respectively), were able to disrupt the integrity of ScV-L-A capsids. The results of this study aid in elucidating the antiviral potential and mechanisms of action of plant-based substances, opening new horizons for the sustainable application of EOs in the control of viral infections. Pronounced antiviral properties-possessing EOs could be applied as natural surface or air sanitizers to control various viruses. Even though they do not immediately exhibit antiviral efficacy, due to their generally recognized safe status, they could be left either on surfaces or vaporized for long periods to provide an additional residual antiviral effect.

## Supplementary Material

Figure S1.jpeg

Supplementary Table S1.docx

## Data Availability

All data supporting the findings of this study are included within the main text of the article and its Supplementary files.
