# Supplementary figures and images for: Essential oils expose diverse targets on non-enveloped ScV-L-A totivirus
Source: Pharm Biol. 2025 Sep 11;63(1):663–82. doi: 10.1080/13880209.2025.2555815 (PMC12427481; doi:10.1080/13880209.2025.2555815)

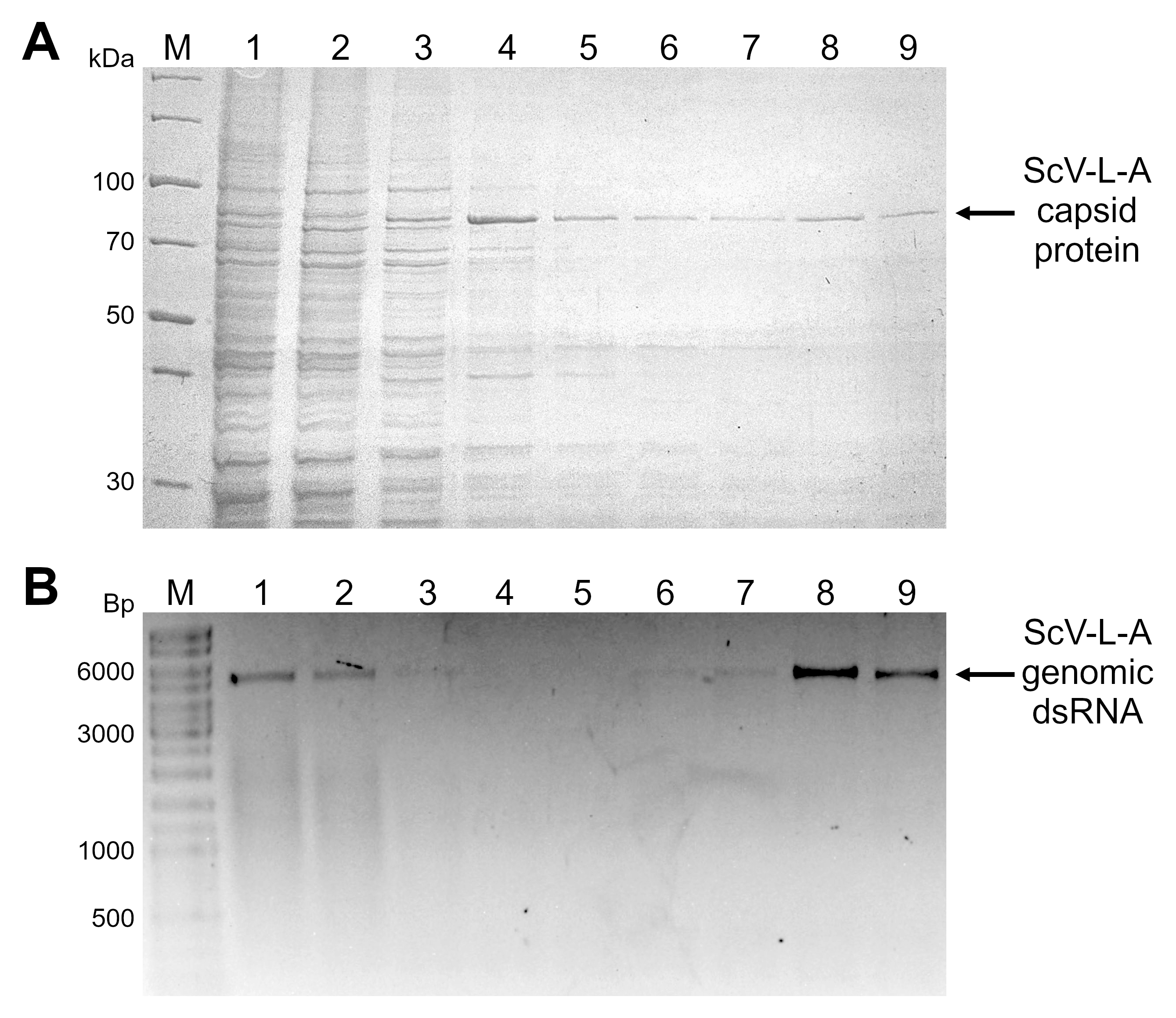

Supplement: Figure S1.jpeg [file IPHB_A_2555815_SM4581.jpeg]
