# Supplementary material for: Essential oils expose diverse targets on non-enveloped ScV-L-A totivirus
Source: Pharm Biol. 2025 Sep 11;63(1):663–82. doi: 10.1080/13880209.2025.2555815 (PMC12427481; doi:10.1080/13880209.2025.2555815)
Supplement: Supplementary Table S1.docx [file IPHB_A_2555815_SM4580.docx]

Table S1. Total composition (%) of essential oils of lemon myrtle (*Backhousia citriodora* F. Muell.), lemongrass (*Cymbopogon citratus* (DC.) Stapf), palmarosa (*Cymbopogon martinii* var. *motia* Bruno), lavender (*Lavandula angustifolia* Mill.), coriander (*Coriandrum sativum* L.), mandarin (*Citrus reticulata* Blanco), lemon verbena (*Aloysia citriodora* Ortega ex Pers.) and tea tree (*Melaleuca alternifolia* Cheel).

| **No** | **Compound** | **RI_Lit._/RI_Exp._** | **Lemon myrtle** | **Lemongrass** | **Palmarosa** | **Lavender** | **Coriander** | **Mandarin** | **Lemon verbena** | **Tea tree** |  |
| --- | --- | --- | --- | --- | --- | --- | --- | --- | --- | --- | --- |
| 1 | Hexanol | 853/855 |  |  |  | 0.08 |  |  |  |  |  |
| 2 | Tricyclene | 927/929 |  | 0.12 |  | 0.01 | 0.03 |  |  |  |  |
| 3 | *α*-Thujene | 930/930 |  |  |  | 0.07 | 0.06 | 0.93 | 0.17 | 0.91 |  |
| 4 | *α*-Pinene | 939/939 |  | 0.19 |  | 0.14 | **7.67** | 2.87 | 1.37 | 2.79 |  |
| 5 | Camphene | 954/955 |  | 1.03 | 0.01 | 0.12 | 1.09 | 0.01 | 0.02 | 0.01 |  |
| 6 | Thuja-2.4(10)-diene | 960/659 |  |  |  |  |  |  | 0.01 |  |  |
| 7 | Sabinene | 975/974 |  | 0.01 |  | 0.03 | 0.37 | 0.27 | 3.19 | 0.16 |  |
| 8 | *β*-Pinene | 979/978 |  | 0.01 |  | 0.07 | 0.64 | 2.14 | 0.23 | 0.76 |  |
| 9 | 1-Octen-3-ol | 979/979 |  |  |  | 0.09 |  |  | 0.04 |  |  |
| 10 | 6-methyl-5-hepten-2-one | 985/985 | 0.26 | 0.98 | 0.02 |  |  |  | 4.34 |  |  |
| 11 | 3-Octanone | 983/983 |  |  |  | 0.50 |  |  |  |  |  |
| 12 | Myrcene | 990/990 |  |  | 0.42 | 0.66 | 1.13 | 2.34 | 0.75 | 0.85 |  |
| 13 | 1.8-*dehydro*-Cineole | 991/991 | 0.55 |  |  |  |  |  |  |  |  |
| 14 | 6-methyl-5-hepten-2-ol | 991/992 |  |  |  |  |  |  | 0.09 |  |  |
| 15 | Butyl butanoate | 994/994 |  |  |  | 0.15 |  |  |  |  |  |
| 16 | 3-Octanol | 991/991 |  |  |  | 0.06 |  |  |  |  |  |
| 17 | (*E*)-*dehydroxy*-Linalool oxide | 993/995 |  |  | 0.01 |  |  |  |  |  |  |
| 18 | *α*-Phellandrene | 1002/1002 |  |  |  | 0.08 | 0.01 | 0.04 | 0.03 | 0.37 |  |
| 19 | 3-*δ*-Carene | 1009/1009 |  |  |  | 0.12 | 0.01 |  |  |  |  |
| 20 | Hexyl acetate | 1012/1012 |  |  |  | 0.26 |  |  |  |  |  |
| 21 | *α*-Terpinene | 1018/1017 |  |  |  | 0.13 | 0.04 | 0.32 | 0.11 | **9.07** |  |
| 22 | *orta*-Cymene | 1026/1024 |  |  |  | 0.08 |  |  |  |  |  |
| 23 | *para*-Cymene | 1024/1024 | 0.03 |  |  | 0.19 | 0.53 | 0.66 | 0.12 | 3.82 |  |
| 24 | Limonene | 1029/1030 |  | 0.27 | 0.17 |  | 2.63 | **67.06** | **22.96** | 1.37 |  |
| 25 | Limonene + *β*-Phellandrene | 1029+1031/1030 |  |  |  | 0.47 |  |  |  |  |  |
| 26 | 1.8-Cineole | 1031/1031 |  |  |  | 0.90 |  |  | **5.37** | **5.98** |  |
| 27 | (Z)-*β*-Ocimene | 1039/1040 |  | 0.25 | 0.42 | 3.88 | 0.01 |  | 0.05 |  |  |
| 28 | (E)-*β*-Ocimene | 1050/1050 |  | 0.14 | 1.62 | 3.30 | 0.01 | 0.02 | 1.42 |  |  |
| 29 | Bergamal | 1050/1051 |  |  |  |  |  |  | 0.11 |  |  |
| 30 | *γ*-Terpinene | 1059/1059 |  |  | 0.01 | 0.12 | **6.82** | **19.50** | 0.29 | **19.23** |  |
| 31 | (*Z*)-Sabinene hydrate | 1070/1071 |  |  |  | 0.06 | 0.08 |  | 0.70 | 0.02 |  |
| 32 | (*Z*)-Sabinene hydrate + (*Z*)-Linalool oxide | 1070+1071/1071 |  |  | 0.01 |  |  |  |  |  |  |
| 33 | 4-Nonanone | 1053/1071 |  | 1.14 |  |  |  |  |  |  |  |
| 34 | (*Z*)-Linalool oxide | 1072/1072 |  |  |  | 0.21 | 0.06 |  |  |  |  |
| 35 | *para*-Mentha-3.8-diene + Unknown | 1072/1073 |  |  |  |  |  | 0.02 |  |  |  |
| 36 | Terpinolene | 1088/1088 |  | 0.04 | 0.02 | 0.05 | 0.60 | 1.03 | 0.08 | 3.23 |  |
| 37 | (*E*)-Linalool oxide | 1086/1088 |  |  |  | 0.14 |  |  |  |  |  |
| 38 | *para*-Cymenene | 1091/1092 |  |  |  |  |  |  |  | 0.11 |  |
| 39 | 1.3.3-Trimethyl-2-ethenyl-cyclohexene | 1095/1094 |  |  |  |  |  |  | 0.30 |  |  |
| 40 | 1.3.8-*para*-Menthatriene | 1110/1100 |  |  |  |  |  |  |  | 0.01 |  |
| 41 | Linalool | 1096/1096 | 0.50 | 0.95 | 2.31 | **24.61** | **67.94** | 0.12 | 0.91 | 0.09 |  |
| 42 | 2.2-Dimethyl-3.4-octadienal | 1098/1103 |  | 0.29 |  |  |  |  |  |  |  |
| 43 | *α*-Pinene oxide | 1099/1103 |  |  |  |  |  |  | 0.42 |  |  |
| 44 | 3-Acetoxy-1-octene | 1103/1106 |  |  |  | 1.81 |  |  |  |  |  |
| 45 | 3-Octanol acetate | 1117/1118 |  |  |  | 0.13 |  |  |  |  |  |
| 46 | Chrysanthemal <cis-> | /1119 | 0.04 |  |  |  |  |  |  |  |  |
| 47 | Fenchol | 1120/1121 |  |  |  |  |  |  | 0.18 |  |  |
| 48 | *allo*-Ocimene | 1121/1121 |  |  | 0.01 | 0.16 |  |  |  |  |  |
| 49 | (*Z*)-*para*-Menth-2-en-1-ol | 1121/1122 |  |  |  |  |  |  |  | 0.15 |  |
| 50 | Butyl tiglate | 1132/1122 |  |  |  | 0.01 |  |  |  |  |  |
| 51 | (*E*)-*para*-Mentha-2.8-dien-1-ol | 1122/1124 | 0.04 |  |  |  |  |  |  |  |  |
| 52 | Campholenal <alpha-> | 1126/1126 |  |  |  |  |  |  | 0.06 |  |  |
| 53 | (*Z*)-Limonene oxide + (*Z*)-*para*-Mentha-2.8-dien-1-ol | 1136+1137/1136 |  |  |  | 0.01 |  |  |  |  |  |
| 54 | (*Z*)-Limonene oxide | 1136/1136 |  |  |  |  |  | 0.04 | 0.14 |  |  |
| 55 | (*Z*)-*para*-Mentha-2.8-dien-1-ol | 1137/1136 | 0.03 |  |  |  |  |  |  |  |  |
| 56 | (*E*)-Limonene oxide | 1142/1140 |  |  |  |  |  | 0.04 |  |  |  |
| 57 | (*E*)-*para*-Menth-2-en-1ol | 1140/1140 |  |  |  |  |  |  |  | 0.12 |  |
| 58 | *neo*-*allo*-Ocimene | 1144/1141 |  |  | 0.01 |  |  |  |  |  |  |
| 59 | Nopinone | 1140/1141 |  |  |  | 0.01 |  |  |  |  |  |
| 60 | (*E*)-Pinocarveol | 1139/1142 |  |  |  | 0.01 |  |  |  |  |  |
| 61 | Camphor | 1145/1145 |  |  |  | 0.34 | 4.07 |  | 0.09 |  |  |
| 62 | (*E*)-Verbenol + Unknown | 1144/1145 |  |  |  |  |  |  | 0.37 |  |  |
| 63 | (*E*)-Chrysanthemol | 1150/1151 |  | 0.25 |  | 0.05 |  |  | 0.50 |  |  |
| 64 | *exo*-Isocitral | 1151/1151 | 0.24 |  |  |  |  |  |  |  |  |
| 65 | Citronellal | 1152/1152 | 0.20 | 0.20 |  |  | 0.04 |  | 0.15 |  |  |
| 66 | Nerol oxide | 1158/1158 |  |  |  | 0.01 |  |  |  |  |  |
| 67 | (*Z*)-Isogeranial | 1164/1163 | 1.62 | 0.87 |  |  |  |  | 0.26 |  |  |
| 68 | *δ*-Terpineol | 1166/1165 |  |  |  |  |  |  | 0.23 |  |  |
| 69 | Pinocarvone | 1164/1164 |  |  |  |  | 0.01 |  |  |  |  |
| 70 | Pinocarvone + Unknown | 1164/1164 |  |  |  | 0.03 |  |  |  |  |  |
| 71 | Lavandulol | 1169/1169 |  |  |  | 1.14 |  |  |  |  |  |
| 72 | Borneol | 1169/1169 |  | 0.30 |  | 1.14 | 0.17 |  |  |  |  |
| 73 | Borneol + *δ*-Terpineol | 1169+1166/1169 |  |  |  |  |  |  |  | 0.02 |  |
| 74 | Rose furan oxide | 1177/1177 |  | 0.19 |  |  |  |  | 0.52 |  |  |
| 75 | Terpinen-4-ol | 1177/1177 |  | 0.04 |  | 4.22 | 0.13 | 0.09 | 0.28 | **40.88** |  |
| 76 | (*E*)-Isogeranial | 1180/1179 | 3.04 | 1.72 |  |  |  |  | 0.52 |  |  |
| 77 | Hexyl isobutyrate | 1157/1180 |  |  |  | 0.29 |  |  |  |  |  |
| 78 | Cryptone | 1185/1183 |  |  |  | 0.15 |  |  |  |  |  |
| 79 | *para*-Cymen-8-ol | 1182/1185 |  |  |  | 0.11 |  |  |  | 0.11 |  |
| 80 | *α*-Terpineol | 1188/1188 |  | 0.19 |  | 0.40 | 0.21 | 0.23 | 1.49 | 3.12 |  |
| 81 | Myrtenal | 1195/1195 |  |  |  |  |  |  | 0.05 |  |  |
| 82 | (*Z*)-Piperitol | 1196/1196 |  | 0.06 |  |  |  |  |  |  |  |
| 83 | Hex-(3Z)-enyl butyrate | 1196/1197 |  |  |  | 0.01 |  |  |  |  |  |
| 84 | *octyl*-Acetate | 1213/1210 |  |  |  | 0.01 |  |  |  |  |  |
| 85 | Decanal | 1201/1200 |  | 0.08 | 0.02 |  | 0.02 | 0.04 |  |  |  |
| 86 | Verbenone | 1205/1205 |  |  |  | 0.03 | 0.01 |  | 0.07 |  |  |
| 87 | (*E*)-Piperitol | 1208/1208 | 0.04 |  |  |  |  |  |  |  |  |
| 88 | Isobornyl formate | 1220/1218 |  |  |  | 0.07 |  |  |  |  |  |
| 89 | (*E*)-Carveol | 1219/1219 |  | 0.05 |  |  |  |  | 0.23 |  |  |
| 90 | Citronellol | 1225/1225 |  |  |  |  | 0.03 |  |  |  |  |
| 91 | (*Z*)-Carveol | 1229/1228 | 0.13 |  |  |  |  |  | 0.12 |  |  |
| 92 | Nerol | 1229/1229 | 0.45 | 0.16 | 0.17 |  |  |  | 0.54 |  |  |
| 93 | Neral | 1238/1238 | **42.35** | **33.82** | 0.25 |  |  |  | **9.80** |  |  |
| 94 | Neral + Hexyl-2-methyl butanoate | 1238+1236/1238 |  |  | 0.03 | 0.12 |  |  |  |  |  |
| 95 | Cuminaldehyde | 1241/1241 |  |  |  | 0.07 |  |  |  |  |  |
| 96 | Carvone | 1243/1243 |  |  |  | 0.05 |  |  | 0.06 |  |  |
| 97 | Linalyl acetate | 1257/1250 |  |  |  | **38.16** |  |  | 0.02 |  |  |
| 98 | Geraniol | 1252/1252 | 1.47 | **8.44** | **70.8** |  | 1.50 |  |  |  |  |
| 99 | Geraniol + Piperitone | 1252+1252/1252 |  |  |  |  |  |  | 0.18 |  |  |
| 100 | 4-Pentenyl hexanoate | 1265/1265 |  |  |  |  |  |  | 0.14 |  |  |
| 101 | Geranial | 1268/1268 | **48.10** | **39.62** |  |  |  |  | **12.56** |  |  |
| 102 | (*E*)-Ascaridol glycol | 1269/1269 |  |  |  |  |  |  |  | 0.06 |  |
| 103 | Perillaldehyde | 1271/1272 |  |  |  |  |  | 0.03 |  |  |  |
| 104 | Phellandral | 1273/1273 |  |  |  | 0.02 |  |  |  |  |  |
| 105 | Lavandulyl acetate | 1290/1290 |  |  |  | 4.55 |  |  |  |  |  |
| 106 | 3-methyl-2-butenyl hexanoate | 1292/1292 |  |  |  |  |  |  | 0.01 |  |  |
| 107 | Cumin alcohol | 1290/1290 |  |  |  | 0.03 |  |  |  |  |  |
| 108 | Thymol | 1290/1290 |  |  |  |  |  | 0.04 | 0.05 |  |  |
| 109 | Geranyl formate | 1298/1298 |  | 0.03 | 0.07 |  |  |  |  |  |  |
| 110 | Carvacrol | 1300/1300 |  |  |  |  |  |  | 0.09 |  |  |
| 111 | Tridecane | 1300/1300 |  |  |  |  | 0.02 |  |  |  |  |
| 112 | Methyl geranate | 1324/1325 |  | 0.04 |  |  |  |  |  |  |  |
| 113 | Myrtenyl acetate | 1326/1326 |  |  |  |  | 0.07 |  |  |  |  |
| 114 | Hexyl tiglate | 1332/1332 |  |  |  | 0.04 |  |  |  |  |  |
| 115 | *δ*-Elemene | 1338/1338 |  |  |  |  |  |  | 0.09 | 0.03 |  |
| 116 | Piperitenone | 1343/1344 |  |  |  |  |  |  | 0.01 |  |  |
| 117 | *α*-Cubebene | 1351/1348 |  |  |  |  |  | 0.01 | 0.05 | 0.04 |  |
| 118 | Eugenol | 1359/1360 |  |  |  |  |  |  | 0.06 |  |  |
| 119 | Neryl acetate | 1361/1361 |  | 0.02 | 0.03 | 0.27 | 0.03 | 0.01 | 0.02 |  |  |
| 120 | Unknown | /1362 |  |  |  |  |  |  | 0.05 |  |  |
| 121 | Cyclosativene | 1371/1372 |  | 0.17 |  |  |  |  | 0.08 |  |  |
| 122 | Isoledene | 1376/1374 |  |  |  |  |  |  |  | 0.06 |  |
| 123 | *α*-Ylangene | 1375/1375 |  | 0.04 |  |  |  |  |  |  |  |
| 124 | *α*-Copaene | 1376/1376 |  | 0.02 |  | 0.02 |  | 0.03 | 0.70 | 0.17 |  |
| 125 | Geranyl acetate | 1381/1380 | 0.23 | 3.63 | **17.76** | 0.44 | 3.89 |  | 1.34 |  |  |
| 126 | 2-*epi*-*α*-Funebrene | 1382/1382 |  |  |  |  |  |  | 0.03 |  |  |
| 127 | *β*-Bourbonene | 1388/1388 |  | 0.04 |  | 0.03 |  |  | 0.48 |  |  |
| 128 | *β*-Cubebene | 1388/1389 |  |  |  |  |  | 0.02 | 0.05 |  |  |
| 129 | 7-*epi*-Sesquithujene | 1391/1390 |  |  |  | 0.04 |  |  |  |  |  |
| 130 | *β*-Elemene | 1390/1390 | 0.09 | 0.17 | 0.15 | 0.02 |  | 0.01 | 0.03 | 0.02 |  |
| 131 | Dodecanal | 1408/1402 |  | 0.02 |  |  |  | 0.02 |  |  |  |
| 132 | Methyl eugenol | 1403/1043 |  |  |  |  |  |  |  | 0.01 |  |
| 133 | Sesquithujene | 1405/1405 |  |  |  | 0.04 |  |  |  |  |  |
| 134 | *α*-Funebrene | 1402/1402 |  |  |  |  |  |  | 0.26 |  |  |
| 135 | *α*-Gurjunene | 1409/1410 |  |  |  |  |  |  | 0.02 | 0.28 |  |
| 136 | *α*-Cedrene +2-*epi*-*β*- Funebrene | 1411+1412/1412 |  |  |  |  |  |  | 0.64 |  |  |
| 137 | Methyl-*N*-methyl-Anthranilate | 1406/1407 |  |  |  |  |  | 0.75 |  |  |  |
| 138 | (*Z*)-Caryophyllene | 1408/1409 |  |  | 0.02 | 0.01 |  |  |  |  |  |
| 139 | *α*-(*Z*)-Bergamotene | 1412/1413 |  |  |  | 0.13 |  |  |  |  |  |
| 140 | *α*-Santalene | 1417/1417 |  |  |  | 0.62 |  |  |  |  |  |
| 141 | (*E*)-Caryophyllene | 1418/1419 | 0.08 | 1.42 | 1.93 | 4.46 | 0.07 | 0.22 | 2.95 | 0.34 |  |
| 142 | *β*-Cedrene | 1420/1420 |  |  |  |  |  |  | 0.23 |  |  |
| 143 | *β*-Copaene | 1432/1432 |  | 0.01 |  |  |  |  | 0.13 |  |  |
| 144 | *α*-(*E*)-Bergamotene | 1434/1434 |  |  |  | 0.22 |  |  | 0.02 |  |  |
| 145 | *γ*-Elemene | 1436/1436 |  | 0.01 |  |  |  |  |  |  |  |
| 146 | Sesquisabinene | 1450/1428 |  |  |  | 0.02 |  |  |  |  |  |
| 147 | *α*-Guaiene | 1439/1440 |  | 0.01 |  |  |  |  |  | 0.06 |  |
| 148 | Aromadendrene | 1440/1440 |  |  |  |  |  |  |  | 1.06 |  |
| 149 | *α*-Maaliene | 1442/1441 |  |  |  |  |  |  |  | 0.06 |  |
| 150 | Isoamyl caprylate | 1445/1445 |  |  | 0.01 |  |  |  |  |  |  |
| 151 | Myltayl-4(12)-ene | 1447/1447 |  |  |  |  |  |  |  | 0.12 |  |
| 152 | Isogermacrene D | 1447/1447 |  |  |  |  |  |  | 0.05 |  |  |
| 153 | (*Z*)-*β*-Farnesene | 1442/1444 |  |  |  | 0.04 |  |  |  |  |  |
| 154 | *epi*-*β*-Santalene | 1447/1449 |  |  |  |  |  |  |  |  |  |
| 155 | (*E*)-*β*-Farnesene | 1452/1452 |  |  | 0.02 | 0.03 |  |  | 0.15 |  |  |
| 156 | (*E*)-Muurola-3.5-diene | 1453/1452 |  | 0.01 |  |  |  |  |  | 0.09 |  |
| 157 | *α*-Humulene | 1454/1454 | 0.01 | 0.40 | 0.14 | 1.85 |  | 0.02 | 0.32 | 0.07 |  |
| 158 | Valerena-4.7(11)-diene | 1455/1455 |  |  |  |  |  |  |  | 0.02 |  |
| 159 | Precocene I | 1463/1461 |  |  | 0.02 |  |  |  |  |  |  |
| 160 | Alloaromadendrene | 1460/1460 | 0.02 |  |  | 0.19 |  |  | 0.68 | 0.43 |  |
| 161 | (*Z*)-Cadina-1(6).4-diene | 1463/1463 |  | 0.02 |  |  |  |  |  |  |  |
| 162 | (*Z*)-Muurola-4(14).5-diene | 1466/1466 |  |  |  | 0.01 |  |  |  |  |  |
| 163 | *β*-Acoradiene | 1470/1471 |  |  |  |  |  |  | 0.20 |  |  |
| 164 | (*E*)-Cadina-1(6).4-diene | 1473/1473 |  | 0.01 |  |  |  |  |  | 0.23 |  |
| 165 | *β*-(*E*)-Bergamotene | 1475/1475 |  |  | 0.02 |  |  |  |  |  |  |
| 166 | Geranyl propanoate | 1477/1477 |  |  |  |  |  |  | 0.27 |  |  |
| 167 | Geranyl propionate + *γ*-Gurjunene + *β*-Chamigrene | 1477/1477 |  |  | 0.10 |  |  |  |  |  |  |
| 168 | *γ*-Muurolene | 1478/1478 |  |  |  |  |  |  | 0.26 | 0.03 |  |
| 169 | *α*-Curcumene | 1480/1480 |  |  |  |  |  |  | 6.77 |  |  |
| 170 | *γ*-Curcumene | 1482/1481 |  |  |  |  |  |  | 0.59 |  |  |
| 171 | Germacrene D | 1481/1482 |  | 0.17 |  | 0.76 |  | 0.01 |  |  |  |
| 172 | *β*-Selinene | 1490/1490 |  |  |  |  |  |  |  | 0.08 |  |
| 173 | *δ*-Selinene | 1492/1492 |  |  |  |  |  |  |  | 0.17 |  |
| 174 | *β*-Selinene + Neryl isobutanoate | 1492/1492 |  |  | 0.04 |  |  |  |  |  |  |
| 175 | (*E*)-Muurola-4(14).5-diene | 1493/1493 |  | 0.02 |  |  |  |  |  |  |  |
| 176 | *epi*-Cubebol | 1494/1495 |  | 0.05 |  |  |  |  |  |  |  |
| 177 | Valencene | 1496/1496 |  |  | 0.05 |  |  | 0.02 |  |  |  |
| 178 | Viridiflorene | 1496/1496 |  |  |  |  |  |  |  | 0.72 |  |
| 179 | *α*-Selinene | 1500/1500 |  |  |  |  |  | 0.12 |  |  |  |
| 180 | Bicyclogermacrene | 1500/1500 | 0.06 |  |  | 0.04 |  |  | 1.34 | 0.17 |  |
| 181 | *α*-Muurolol | 1500/1500 |  | 0.02 |  |  |  |  |  | 0.11 |  |
| 182 | (*E*.*E*)-*α*-Farnesene | 1505/1505 |  |  | 0.01 |  |  | 0.46 |  |  |  |
| 183 | *β*-Himachalene | 1500/1506 | 0.02 |  |  |  |  |  |  |  |  |
| 184 | *α*-Chamigrene + Unknown | 1503/1506 |  | 0.04 |  |  |  |  |  |  |  |
| 185 | *β*-Bisabolene | 1507/1507 |  |  | 0.01 | 0.04 |  |  | 0.15 |  |  |
| 186 | *β*-Curcumene | 1515/1515 |  |  |  |  |  |  | 0.69 |  |  |
| 187 | *γ*-Cadinene | 1512/1513 |  | 1.07 | 0.03 | 0.17 |  |  | 0.26 | 0.02 |  |
| 188 | Teresantalol | /1517 |  |  |  | 0.15 |  |  |  |  |  |
| 189 | Cubebol | 1515/1515 |  |  |  |  |  |  | 0.23 |  |  |
| 190 | 7-*epi*-*α*-Selinene | 1522/1522 |  |  | 0.02 |  |  |  |  |  |  |
| 191 | *δ*-Cadinene | 1523/1523 | 0.02 | 0.20 | 0.02 |  |  | 0.03 | 0.43 | 1.08 |  |
| 192 | (*E*)-*γ*-Bisabolene | 1528/1528 |  | 0.11 |  |  |  |  |  |  |  |
| 193 | (*Z*)-Nerolidol | 1531/1530 |  |  | 0.02 |  |  |  |  |  |  |
| 194 | (*E*)-Cadina-1.4-diene | 1534/1535 |  | 0.04 |  |  |  |  | 0.06 | 0.16 |  |
| 195 | *α*-Cadinene | 1538/1538 |  | 0.03 |  |  |  |  | 0.07 | 0.01 |  |
| 196 | *α*-Calacorene <alpha-> | 1545/1545 |  |  |  |  |  |  | 0.02 |  |  |
| 197 | *α*-Elemol | 1549/1549 |  | 0.05 |  |  |  |  |  |  |  |
| 198 | (*E*)-Nerolidol | 1563/1563 |  |  | 0.17 |  |  |  | 0.71 |  |  |
| 199 | Germacrene B | 1561/1560 | 0.03 |  |  |  |  |  |  |  |  |
| 200 | Germacrene B + (*Z*)-Muurola-5-en-4-alpha-ol | 1561+1551/1560 |  |  |  |  |  |  | 0.05 |  |  |
| 201 | Geranyl butyrate | 1564/1564 |  | 0.12 | 0.34 |  |  |  |  |  |  |
| 202 | Epiglobulol | 153/1565 |  |  |  |  |  |  |  | 0.06 |  |
| 203 | Spathulenol | 1578/1578 | 0.04 |  |  |  |  |  | 2.18 | 0.06 |  |
| 204 | Caryophyllene oxide | 1583/1583 | 0.03 | 0.33 | 0.19 | 0.31 |  | 0.01 | 2.61 |  |  |
| 205 | Viridiflorol | 1592/1594 |  |  | 0.05 |  |  |  |  | 0.26 |  |
| 206 | Cubeban-11-ol | 1595/1595 |  |  |  |  |  |  |  | 0.21 |  |
| 207 | Rosifoliol | 1600/1600 |  |  |  |  |  |  |  | 0.08 |  |
| 208 | Cedrol | 1600/1600 |  |  |  |  |  |  | 0.13 |  |  |
| 208 | Humulene epoxide II | 1608/1608 |  | 0.03 | 0.01 |  |  |  | 0.32 |  |  |
| 209 | 1.10-Diepicubenol | 1618/1619 |  | 0.01 |  |  |  |  | 0.03 |  |  |
| 210 | Epicubenol | 1632/1632 |  | 0.02 |  |  |  |  | 0.03 |  |  |
| 211 | Caryophylla-4(12).8(13)-dien-5-*β*-ol | 1636/1640 |  |  | 0.01 |  |  |  |  |  |  |
| 212 | *epi*-*α*-Cadinol | 1640/1640 |  |  |  | 0.09 |  |  | 0.55 |  |  |
| 213 | Cubenol | 1644/1645 |  |  |  |  |  |  |  | 0.13 |  |
| 214 | T-Muurolol + *α*-Muurolol | 1642+1646/1645 |  | 0.02 |  |  |  |  |  |  |  |
| 215 | *β*-Eudesmol | 1650/1650 |  |  |  |  |  |  |  | 0.09 |  |
| 216 | *α*-Cadinol <alpha-> | 1654/1654 |  |  |  |  |  |  | 0.20 |  |  |
| 217 | *α*-Eudesmol | 1653/1655 |  |  |  |  |  |  | 0.05 |  |  |
| 218 | Allohimachalol | 1662/1663 |  |  |  |  |  |  | 0.11 |  |  |
| 219 | Germacra-4(15).5.10(14)-trien-1-*α*-ol | 1686/1686 |  |  |  |  |  |  | 0.05 |  |  |
| 220 | Acorenone B | 1697/1696 |  |  |  |  |  |  | 0.06 |  |  |
| 221 | Geranyl hexanoate | 1725/1725 |  |  | 1.50 |  |  |  |  |  |  |
| 222 | (2*E*.6*E*)-Farnesol | 1746/1745 |  |  | 0.40 |  |  |  |  |  |  |
| 223 | *α*-Sinensal <alpha-> | 1756/1756 |  |  |  |  |  | 0.36 |  |  |  |
| 224 | Neophytadiene | 1830/1830 |  | 0.02 | 0.04 |  |  |  |  |  |  |
| 225 | Farnesyl acetate | 1845/1845 |  |  | 0.15 |  |  |  |  |  |  |
| 226 | Phytone | 1851/1852 |  | 0.01 | 0.06 |  |  |  | 0.06 |  |  |
| 227 | Geranyl octanoate | 1928/1929 |  | 0.01 | 0.29 |  |  |  |  |  |  |
| 228 | Phytol | 2114/2112 |  |  |  |  |  |  | 0.02 |  |  |
|  | **Total** |  | **99.72** | **99.85** | **99.99** | **99.74** | **100** | **99.94** | **98.73** | **99.77** |  |
